# Association between attendance at an American diabetes camp and improvements in glycaemic control and treatment satisfaction

**DOI:** 10.1002/edm2.254

**Published:** 2021-05-04

**Authors:** Amy Darukhanavala, Sarah Puhr, Kyle Dinunno, David Alfego, John Welsh, Lynn Butler, Kendra Magyar

**Affiliations:** ^1^ Division of Pediatric Endocrinology UMass Medical Center Worcester MA USA; ^2^ Dexcom, Inc San Diego CA USA; ^3^ The Barton Center for Diabetes Education North Oxford MA USA

**Keywords:** continuous glucose monitoring, diabetes camp, glycaemic control, quality of life

## Abstract

**Introduction:**

Few studies have evaluated glycaemic control using continuous glucose monitoring (CGM) in individuals before and after attendance at a diabetes camp or by comparing control groups at home to control groups at camp.

**Methods:**

Youth (6–17 years) with T1D and receiving insulin therapy were enrolled at a week‐long diabetes camp. They participated in three clinic visits: at the start of a week at home, by initiating a Dexcom G6 CGM system; at the start of a week at camp, where the home week G6 was removed and a camp week G6 was inserted; and after camp, where the camp week G6 was removed. We administered Problem Areas in Diabetes (PAID) surveys at the second and third visits. Participants with <80% CGM data coverage or who did not complete all PAID surveys were excluded from analysis. We compared glycaemic control and PAID scores between the week at home and week at camp.

**Results:**

Of 76 enrolled campers, 69 completed the study and 52 had results that qualified for analysis. The mean participant age was 12.5 ± 2.2 years. Camp was associated with significantly improved treatment satisfaction, time in desired glucose range and insulin sensitivity. Time in hyperglycaemia and basal insulin requirements decreased significantly.

**Conclusions:**

Diabetes camp is associated with significant improvements in diabetes treatment satisfaction and glycaemic control compared to home care.

## Graphical abstract

Children with Type I Diabetes enjoying fun and safe activities at Barton Diabetes Camp. Glycemic control and satisfaction with diabetes improved during camp weeks.

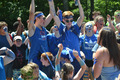

## 1 INTRODUCTION

Diabetes camps provide motivation, education and a long‐lasting support network for children and adolescents with type 1 diabetes (T1D). Some camps also expose participants to modern diabetes management technologies, such as continuous glucose monitoring (CGM) and automated insulin delivery (AID) systems, either through investigator‐initiated studies or interactions with other campers. The opportunity to learn therapeutic strategies in a positive setting with peers is effective^1^ resulting in improved self‐care skills,^2^ quality of life,^2^, ^3^, ^4^ and haemoglobin A1c (HbA1c) levels.^5^ However, few studies have examined whether glycaemic control at home prior to camp is different than that during camp. Additionally, previous studies have used point‐of‐care tests to measure glycaemic control. Studies using CGM systems are limited but valuable, because CGM data are quantitatively and qualitatively better than point‐estimate finger stick glucose measurements or HbA1c. CGM data accurately assess the amount of time spent in the glucose target range of 70–180 mg/dl, also called ‘time in range’ (TIR), and the time above or below this range in different settings, such as at home versus at camp. To our knowledge, no studies have examined these differences. We utilized an accurate, factory‐calibrated, real‐time CGM system to compare glycaemic control in home and camp settings.

## 2 METHODS

This prospective, non‐randomized, observational study of youth ages 6–17 years was conducted at Barton Center week‐long residential camps. Study participants were recruited from registrants for the Summer 2019 session. Inclusion criteria included English fluency and T1D managed with intensive insulin therapy (multiple daily injections or continuous subcutaneous insulin infusion). Exclusion criteria included type 2 diabetes (T2D) or use of an AID system; participants could use predictive low‐glucose suspend insulin pump systems. The study was approved by a central institutional review board, and we obtained written informed consent. All procedures were in accordance with the ethical standards of the institutional review board and ethics committee on human experimentation (institutional and national) and with the Helsinki Declaration of 1964, as revised in 2013.

The study included three visits with a Barton Center study team member. During the first clinic visit at the start of ‘home week’, we reviewed participant eligibility, obtained informed consent, recorded the participant's most recent HbA1c measurement and initialized a new Dexcom G6 CGM system (G6; Dexcom) with sensor, transmitter and receiver. CGM data were visualized on the new receiver. Participants chose whether to enable low or high threshold alerts or the Urgent Low Soon predictive alert during home use. If participants used alerts at home, they were asked to use the same settings for camp. During the second clinic visit at the start of ‘camp week’, we removed the home week G6 CGM, initialized a camp week G6 CGM and administered a post‐home‐week Problem Areas in Diabetes (PAID) questionnaire. The PAID survey is 20‐item scale with each item scored from 0 (not a problem) to 4 (serious problem); higher total scores reflect greater emotional distress. During the third clinic visit at the end of camp week, we removed the camp week G6 CGM, downloaded the CGM data from the transmitter and administered a post‐camp week PAID questionnaire.

During camp week, we set the G6 system to alarm when glucose concentrations were ≤55 mg/dl or ≥350 mg/dl and all systems were centrally monitored during the day and night by healthcare staff members. When glucose levels were outside the 55–350 mg/dl range in the daytime or outside the 80–300 mg/dl range at night‐time, counsellors or medical staff performed confirmation finger stick glucose measurements and interventions such as blood ketone testing for hyperglycaemia. Otherwise, camp staff used G6 CGM data to inform treatment decisions.

Campers who had <80% CGM data coverage (predominantly due to loss of connectivity) or who did not complete all PAID questionnaires were excluded from the analysis. We compared satisfaction scores on PAID questionnaires and CGM‐derived glycaemic measurements, including time spent in hypoglycaemia level 1 (<70 mg/dl) or level 2 (<54 mg/dl), TIR and time spent in hyperglycaemia level 1 (>180 mg/dl) or level 2 (>250 mg/dl), between the home week and camp week. The Shapiro‐Wilk test was used to determine whether data were distributed normally. We analysed normally distributed data with a paired Student's *t*‐test and non‐normally distributed data with the Wilcoxon signed rank test.

## 3 RESULTS

Of 76 study‐enrolled campers, 69 completed the study, and 52 provided adequate data. Analysed participants had a mean age of 12.5 ± 2.2 years (range 7–16) and were 59.6% female, 38.5% male and 1.9% unidentified. The majority (92.3%) of participants identified as Non‐Hispanic White. Most participants were familiar with diabetes technologies: 82.7% used insulin pumps and 86.6% routinely used CGM. Pre‐camp HbA1c values from 49 participants averaged 7.7 ± 1.1%. Three participants did not have available HbA1c values. During the home week, mean average glucose was 189.1 ± 37.9 mg/dl, with no major difference between younger children (6–12 years; 189.6 ± 36.4 mg/dl) and adolescents (13–17 years; 188.6 ± 39.9 mg/dl).

Most measures of glycaemic control improved significantly from home week to camp week. Mean TIR increased from 49.5% ± 20.1% to 67.1% ± 29.5% (*p* < .01) (Table 1). Time in level 1 and level 2 hyperglycaemia decreased dramatically; time in level 2 hyperglycaemia fell by more than half at camp (21.7$ ± 16.9% vs. 10.1$ ± 8.3%, *p* < .01). Similarly, mean glucose measures significantly decreased from 189.1 ± 37.9 mg/dl during home week to 155.8 ± 25.8 mg/dl during camp week (*p* < .01). The average glucose at camp was similar between younger children (159.4 ± 26.3 mg/dl) and adolescents (152.8 ± 25.5 mg/dl). There was an increase in hypoglycaemia at camp. Percent time spent in level 1 hypoglycaemia increased significantly from 2.0% ± 2.4% to 3.3% ± 2.9% (*p* < .01), whereas time spent in level 2 hypoglycaemia increased from 0.4% ± 0.7% to 0.6% ± 1.0% (*p* < .08). Hypoglycaemia increased despite decreases in daily insulin usage: basal insulin totals decreased from 23.7 ± 13.9 U/day at home to 18.8 ± 11.4 U/day at camp (*p* < .01). Insulin boluses decreased due to a modest increase in insulin to carbohydrate ratios (1:13 at home vs. 1:14.2 at camp, *p* < .01). No severe hypoglycaemic events occurred during the study.

**TABLE 1:** Difference in continuous glucose monitoring‐derived glycemic metrics between home and camp weeks, mean ± SD, *N* = 52

	Home week	Camp week	*p*‐Value
Mean sensor Glucose (mg/dl)	189.1 ± 37.9	155.8 ± 25.8	<.01
Percent time spent <54 mg/dl (<3.0 mmol/L) (%)	0.4 ± 0.7	0.6 ± 1.0	.08
Percent time spent <70 mg/dl (3.9 mmol/L) (%)	2.0 ± 2.4	3.3 ± 2.9	<.01
Percent time spent in Range, 70–180 mg/dl (3.9–10.0 mmol/L) (%)	49.5 ± 20.1	67.1 ± 29.5	<.01
Percent time spent >180 mg/dl (>10.0 mmol/L) (%)	48.5 ± 20.7	29.5 ± 15.2	<.01
Percent time spent >250 mg/dl (>13.9 mmol/L) (%)	21.7 ± 16.9	10.1 ± 8.3	<.01

Quality of life, as measured by PAID questionnaire, improved significantly from 30.3 ± 18.9 at home to 23.2 ± 16.2 at camp (*p* < .01). Of the subjects with high distress scores (≥40) at the end of home week (*N* = 16), ten campers improved their score from 48.8 ± 8.6 to 27.6 ± 11.4 at the end of camp week, five campers improved their score from 63.3 ± 12.6 to 49.0 ± 8.8 (remained a high distress score at the end of camp week) and one camper had no change in score 42.5–42.5. Only four campers worsened their scores between post home week 31.3 ± 4.9 to a high distress score post‐camp week 46.9 ± 1.6.

## 4 DISCUSSION

Diabetes camp is a unique opportunity for youth with diabetes to build friendships, overcome isolation and become empowered to manage diabetes. Camp can also foster development and dissemination of technology, such as AID systems.^6^ Diabetes camp attendance is associated with improved glycaemic control.^5^, ^7^, ^8^ However, previous studies have compared glycaemic control at camp to that from time points 3–12 months later and have relied on HbA1c measurements or point‐of‐care finger stick testing. Few studies have used CGM at camp to evaluate changes in glycaemic control immediately before and during camp.

This study demonstrates that CGM is feasible at diabetes camp and that most CGM‐derived measures of glycaemic control are significantly better at camp than at home. In particular, youth at camp are close to meeting consensus recommendations for TIR, despite being part of an age group that is typically refractory to treatment.^9^, ^10^ Although we found an increase in hypoglycaemia at camp consistent with previous studies,^11^, ^12^ campers in our study met consensus goals for time spent below glucose target range. Additionally, hyperglycaemia decreased by more than 50% at camp compared to a home setting, without requiring increases in basal or bolus insulin doses. A previous study found that CGM reduced hypoglycaemia at camp better than finger stick testing^13^; however, we did not perform finger stick testing to compare between home and camp settings and cannot compare our results to that study.

Interestingly, some American diabetes camps are reluctant to incorporate CGM systems into their protocols due to concerns about liability, equipment loss, alarms disturbing camper sleep and the desire to ‘liberate’ campers from diabetes. To address the recognized increase in hypoglycaemic episodes at camp, setting a higher low threshold alert on the CGM may allow camp staff to intervene prior to the onset of clinical hypoglycaemia. In our study, use of the G6’s remote monitoring feature was precluded; however, camps that have implemented remote monitoring of CGM data observed significant reductions in hypoglycaemia, particularly overnight.^14^, ^15^

Our study contributes to a body of evidence^4^, ^5^ indicating that diabetes camps reduce diabetes‐related distress, promote short‐term improvements in glycaemic control and enhance quality of life. Additional research is required to show whether CGM‐measured glycaemic benefits are sustained after camp. This study is limited by the absence of a control group who wore blinded CGM before camp or a control group who did not attend camp. Thus, further studies are needed to determine whether diabetes camp or CGM use was responsible for the improved glycaemic control observed. Also, the study population included a greater proportion of CGM and insulin pump users compared to respondents of the T1D Exchange,^9^ and participants also had a lower mean HbA1c value than expected for their age range. These characteristics suggest a higher level of treatment engagement among Camp Barton participants. Results from Camp Barton may not be generalizable to other camps with different diabetes management strategies.

## CONFLICT OF INTEREST

Dr. Sarah Puhr and Dr. John Welsh are employed by Dexcom Inc. All other authors have no conflict of interest to report.

## AUTHOR CONTRIBUTIONS

A.D., K.D, L.B and K.M. performed the research and designed the research study. D.A. analysed the data. S.P., J.W. and A.D. wrote the paper.

## Supporting information

Data S1

## Data Availability

The data that support the findings of this study are available on request from the corresponding author. The data are not publicly available due to privacy or ethical restrictions.
