# Supplementary material for: Association between attendance at an American diabetes camp and improvements in glycaemic control and treatment satisfaction
Source: Endocrinol Diabetes Metab. 2021 May 4;4(3):e00254. doi: 10.1002/edm2.254 (PMC8279631; doi:10.1002/edm2.254)
Supplement: Supplementary file 1 — Data S1 [file EDM2-4-e00254-s001.docx]

| **DEMOGRAPHICS** |  |
| --- | --- |
| **N** | 52 |
| **Avg Age** | 12.5 ± 2.2 |
| **Sex (%)** |  |
| ***F*** | 31 (59.6%) |
| ***M*** | 20 (38.5%) |
| ***N*** | 1 (1.9%) |
| **Ethnicity (%)** |  |
| ***Non-Hispanic or Latino*** | 49 (94.2%) |
| ***Hispanic or Latino*** | 1 (1.9%) |
| ***Unknown*** | 2 (3.9%) |
| **Race (%)** |  |
| ***White*** | 48 (92.3%) |
| ***Black or African American*** | 2 (3.9%) |
| ***>1 Race*** | 1 (1.9%) |
| ***American Indian / Alaska Native*** | 1 (1.9%) |
| **Pump/Injection (%)** |  |
| ***Omnipod*** | 27 (51.9%) |
| ***Tslim*** | 9 (13.5%) |
| ***670G*** | 5 (9.6%) |
| ***Lantus + Humalog*** | 4 (7.7%) |
| ***Minimed Revel*** | 2 (3.9%) |
| ***Basaglar + Novolog*** | 1 (1.9%) |
| ***Injections*** | 1 (1.9%) |
| ***Minimed 630*** | 1 (1.9%) |
| ***Lantus + Apidra*** | 1 (1.9%) |
| ***Tslim basal IQ*** | 1 (1.9%) |
| ***Tslim + Tresiba*** | 1 (1.9%) |
| ***Tresiba + NPH Novolog*** | 1 (1.9%) |
| **CGM (%)** |  |
| ***Dexcom G6*** | 40 (76.9%) |
| ***None*** | 7 (13.5%) |
| ***Dexcom G5*** | 3 (5.8%) |
| ***Dexcom G4*** | 1 (1.9%) |
| ***Guardian*** | 1 (1.9%) |
| **Avg Last A1c** | 7.7 ± 1.1 |

Data Set:

PAID SCORES:
